# Restless Legs Syndrome: A Network Model of Iron-Dependent Neuromodulation—A Narrative Review

**DOI:** 10.3390/brainsci16050440

**Published:** 2026-04-22

**Authors:** Oscar Arias-Carrión

**Affiliations:** 1 División de Neurociencias Clínica, Instituto Nacional de Rehabilitación Luis Guillermo Ibarra Ibarra, Mexico City 14389, Mexico; ariasemc2@gmail.com; 2 Tecnologico de Monterrey, Escuela de Medicina y Ciencias de la Salud, Mexico City 14380, Mexico

**Keywords:** restless legs syndrome, brain iron dysregulation, augmentation, dopaminergic plasticity, adenosinergic dysfunction, circadian neuromodulation, network neuroscience

## Abstract

**Highlights:**

**What are the main findings?**

- Restless legs syndrome is reframed as an iron-dependent disorder of distributed neuromodulatory instability, in which altered brain iron availability disrupts dynamic interactions across cortico–striatal–thalamo–limbic circuits, extending beyond a dopamine-centric paradigm toward a network-based model of state-dependent dysfunction.
- An integrative, multi-layered framework is proposed in which brain iron dysregulation, polygenic susceptibility, and circadian modulation converge to destabilize dopaminergic, adenosinergic, glutamatergic, and noradrenergic signalling, driving network hyperexcitability, phenotypic variability, and treatment-related augmentation.

**What are the implications of the main findings?**

- These findings support a shift toward early, trajectory-modifying strategies, prioritizing timely identification, targeted iron repletion, and restrained dopaminergic exposure to reduce augmentation, restore sleep–wake stability, and limit neuropsychiatric burden.
- The model provides a foundation for biologically informed stratification of RLS, enabling the delineation of mechanistically distinct subtypes (e.g., iron-driven, circadian-dominant, hyperexcitability-predominant), while underscoring the need for longitudinal, multimodal validation to translate this framework into precision clinical practice.

**Abstract:**

Restless legs syndrome (RLS) is traditionally conceptualized as a dopamine-responsive sensorimotor disorder; however, new evidence suggests a more complex and heterogeneous neurobiological basis. Findings from neuroimaging, genetic studies, circadian biology, and clinical research indicate that dopaminergic dysfunction occurs within a broader context of neuromodulatory imbalance involving iron metabolism, adenosinergic signalling, glutamatergic excitability, and, potentially, noradrenergic pathways. In parallel, quantitative susceptibility mapping and related approaches have provided indirect evidence of altered brain iron distribution, although results remain variable across studies. Clinically, RLS extends beyond nocturnal discomfort and is associated with sleep fragmentation, impaired quality of life, and neuropsychiatric comorbidity, as well as treatment-related complications such as augmentation. However, current diagnostic frameworks remain predominantly phenomenological, and available biomarkers lack sufficient validation for routine clinical use. In this narrative review, the available clinical, genetic, and neuroimaging evidence is synthesized to propose an integrative, network-based model in which iron-dependent neuromodulatory processes influence excitability across cortico–striatal–thalamo–limbic circuits. This framework is intended as a hypothesis-generating model rather than a definitive explanation of disease mechanisms. Substantial heterogeneity across studies, together with variability in clinical presentation and limited reproducibility of candidate biomarkers, underscores the need for standardized methodologies and longitudinal, multimodal investigations. Future work should aim to test this model empirically, refine biological stratification, and determine whether network-informed approaches can improve diagnosis and therapeutic targeting in RLS.

## 1. Introduction

Restless legs syndrome (RLS) is a common sleep-related movement disorder that remains poorly defined in neurology. It is prevalent, often distressing, and frequently treatable. Nevertheless, despite these features, it is still widely under-recognized and undertreated in clinical practice. Traditionally, RLS has been framed as a benign sensorimotor condition, largely defined by its hallmark clinical feature: an urge to move the legs, typically emerging or worsening in the evening and at rest, and showing responsiveness to dopaminergic therapies [1,2,3]. This dopamine-centred framework has been supported by robust clinical and pharmacological evidence, including symptomatic improvement with dopamine agonists and insights from functional imaging studies. However, it does not fully account for the disorder’s complexity, variability, and long-term trajectory.

Evidence over the past decade has expanded, rather than replaced, this perspective, suggesting that RLS may be more appropriately understood as a disorder of iron-dependent neuromodulation embedded within distributed cortico–striatal–thalamo–limbic networks. Convergent findings from neuroimaging, cerebrospinal fluid analysis, genetics, and longitudinal epidemiology indicate that dopaminergic dysfunction occurs within a broader network context, shaped by iron handling at the blood–brain barrier, circadian dopaminergic dynamics, glutamatergic hyperexcitability, and endogenous opioid modulation [1,2,3]. In addition, new evidence implicates adenosinergic dysfunction, particularly adenosine A1 receptor downregulation, as a potential mediator linking iron deficiency to increased glutamatergic tone and network hyperexcitability. In addition to these systems, emerging evidence suggests that noradrenergic (adrenergic) pathways may contribute to the pathophysiology of RLS. The locus coeruleus, a principal source of central noradrenergic projections, plays a critical role in arousal regulation, sensory processing, and modulation of spinal motor circuits [4,5]. Dysregulation of adrenergic tone may contribute to the hyperarousal state, sensory amplification, and motor restlessness characteristic of RLS, particularly in the context of sleep–wake instability [6].

Globally, RLS affects up to 5% of adults, with higher prevalence among women and older individuals. Nevertheless, underdiagnosis remains common, particularly in primary care settings, where symptoms are often misattributed to neuropathy, anxiety, insomnia, or ageing [1,2,3]. More importantly, RLS is not confined to discomfort. It is associated with chronic sleep fragmentation, depressive syndromes, and impulse control disorders under dopaminergic exposure, while associations with broader systemic outcomes, including cardiovascular risk, remain an area of active investigation rather than established causality [7]. These observations suggest that RLS extends beyond a motor restlessness syndrome toward a disorder of neurobiological regulation with multisystem implications.

Mechanistically, central iron deficiency has emerged as a reproducible biological substrate. Advanced MRI techniques such as quantitative susceptibility mapping demonstrate reduced iron content in the substantia nigra and related circuits despite normal peripheral ferritin levels [1,8]. Genome-wide association studies have identified more than 150 risk loci, implicating genes such as *MEIS1* and BTBD9, which link neurodevelopmental programming to iron homeostasis and dopaminergic regulation [9,10]. These findings support a developmental–metabolic vulnerability model in which genetically primed circuits become destabilized under conditions of fluctuating iron availability, hormonal changes, systemic inflammation, or ageing.

The therapeutic implications are complex. Although dopamine agonists improve symptoms in the short term, long-term exposure carries a predictable risk of augmentation, a paradoxical worsening of disease associated with maladaptive plasticity within dopaminergic pathways. Current guidelines prioritize iron repletion and α2δ ligands over dopaminergic agents for chronic therapy [11]. This shift reflects an evolving recognition that dopaminergic dysfunction represents one component of a broader network disturbance, rather than a singular causal mechanism.

The field now stands at an inflection point. Three conceptual transitions are emerging. First, from phenomenology to biology: diagnosis remains clinical, yet emerging biomarkers, including brain iron imaging, polygenic risk profiling, and circadian neurochemical signatures, offer the potential for biologically stratified subtypes, though these tools remain under validation. Second, from symptomatic control to potential disease modification: early correction of iron insufficiency and avoidance of chronic dopaminergic overexposure may influence disease trajectories, but this hypothesis requires confirmation in longitudinal studies. Third, from isolated disorder to systemic syndrome: RLS intersects with mood disorders, chronic kidney disease, pregnancy-related iron shifts, and neurodegenerative conditions, suggesting a broader network vulnerability influenced by systemic and developmental factors.

Within this context, RLS can be conceptualized as a network disorder in which iron availability modulates interactions among dopaminergic, adenosinergic, and glutamatergic systems, leading to altered excitability across motor, sensory, and limbic circuits. This integrative perspective provides a mechanistic basis for clinical heterogeneity, treatment response variability, and the phenomenon of augmentation.

In this narrative review, we synthesize advances in pathophysiology, genetics, neuroimaging, clinical presentation, comorbidities, and treatment to propose an integrative, hypothesis-generating framework that extends beyond dopamine-centric models toward an iron-informed, network-based understanding of RLS. RLS is not simply a syndrome of restless legs. It represents a model through which subtle metabolic and neuromodulatory disturbances can destabilize neural circuits governing movement, sleep, and affect. Future progress will depend on integrating multimodal evidence, refining biological stratification, and validating mechanistic targets, rather than relying solely on symptom-based approaches.

## 2. Search Strategies

This study was designed as a structured narrative review from the outset, aiming to provide an integrative, hypothesis-generating synthesis of the literature on RLS. This approach was selected to allow conceptual integration across heterogeneous domains, including pathophysiology, epidemiology, clinical presentation, diagnosis, and management, which would not be adequately captured through a strictly systematic framework.

Electronic searches were conducted in PubMed/MEDLINE, Scopus, and Web of Science from database inception through November 2025. The search strategy combined controlled vocabulary terms (e.g., MeSH) and free-text keywords related to RLS and its underlying mechanisms. A representative PubMed query included: (“restless legs syndrome” OR “Willis–Ekbom disease”) AND (“iron” OR “brain iron deficiency” OR “dopamine” OR “adenosine” OR “glutamate” OR “circadian”) AND (“genetics” OR “*MEIS1*” OR “BTBD9” OR “GWAS” OR “neuroimaging” OR “MRI” OR “treatment” OR “gabapentinoids” OR “dopamine agonists” OR “augmentation”). Additional terms included “periodic limb movements,” “iron therapy,” and “sleep disorders.” Reference lists of relevant reviews, clinical guidelines, and primary studies were manually screened to identify additional articles.

The initial search identified approximately 1200–1500 records. After title and abstract screening, studies were assessed at the full-text level, and approximately 180–220 articles were incorporated into the final synthesis. Given the narrative design, formal PRISMA flow diagrams and quantitative pooling were not used.

Importantly, study selection was performed in a section-specific manner to align evidence with the conceptual focus of each part of the manuscript. For the epidemiology section, priority was given to population-based studies, large cohort analyses, and meta-analyses reporting prevalence, sex differences, and age-related trends. For clinical presentation and systemic implications, studies describing symptom phenotypes, sleep disruption, neuropsychiatric associations, and comorbidities were prioritized, including both observational cohorts and polysomnographic investigations. For pathophysiology and mechanisms, emphasis was placed on neuroimaging studies, genetic analyses (including genome-wide association studies), and experimental or translational research addressing iron metabolism, dopaminergic and adenosinergic signalling, and network-level dysfunction. For diagnostic approaches, clinical criteria papers, guideline statements, and studies evaluating biomarkers or ancillary testing were prioritized. For treatment and management, randomized clinical trials, meta-analyses, and guideline-based recommendations were preferentially included.

Across all sections, priority was given to recent systematic reviews, meta-analyses, randomized clinical trials, large cohort studies, genome-wide association studies, and neuroimaging investigations that provided mechanistic or clinically actionable insights. Seminal earlier studies were included where necessary to ensure conceptual continuity. Evidence from major consensus statements and clinical practice guidelines, including those from the International Restless Legs Syndrome Study Group and the American Academy of Sleep Medicine (AASM), was incorporated when relevant to diagnostic and therapeutic recommendations.

Study selection was guided by predefined qualitative criteria, including methodological rigour, reproducibility of findings, adequacy of sample size, and relevance to clinically or biologically meaningful questions. Rather than selecting studies to support a single hypothesis, emphasis was placed on integrating converging and, where applicable, conflicting lines of evidence across independent methodologies.

Articles not available in English, case reports with limited generalisability, and studies lacking methodological transparency were generally excluded, unless they provided unique mechanistic insights. A formal risk-of-bias assessment was not performed because it is not standard for narrative reviews. Instead, conclusions were based on consistency across multiple independent studies rather than single reports. This approach enabled integration of molecular, imaging, genetic, and clinical evidence while acknowledging the inherent limitations of narrative methodologies, including potential selection bias and reduced reproducibility compared with systematic reviews.

## 3. Epidemiology of RLS: Prevalence, Sex Differences, and the Challenge of Under-Recognition

RLS is a common neurological condition that is often not recognized in clinical practice. Population studies estimate a global prevalence of 1.9% to 4.6% [12], although rates vary depending on diagnostic criteria, population characteristics, and study design. In the United States, about 8% of adults report symptoms suggestive of RLS within a given year, while approximately 3% have symptoms that are frequent and severe enough to affect sleep and quality of life [12]. These figures place RLS among the most common movement disorders, yet its true burden is likely underestimated.

A key feature of RLS is the gap between symptom onset and clinical diagnosis. In nearly two-thirds of patients, symptoms begin before the age of 45 [13]. However, many individuals do not seek medical attention until later in life, often in their fifth or sixth decade. This delay reflects the intermittent and initially mild nature of symptoms, as well as their tendency to be attributed to other conditions such as stress, insomnia, peripheral neuropathy, or normal ageing. As a result, RLS often remains unrecognized for many years.

The prevalence of RLS increases with age, affecting around 10% of adults aged 65 years and older [14]. Women are consistently affected more often than men, with a ratio of approximately 2:1 in older populations [14]. Several factors may contribute to this difference, including hormonal influences, changes in iron levels during pregnancy, and possible genetic susceptibility. Iron metabolism has emerged as an important factor in RLS, particularly given its role in brain function, although the exact mechanisms remain unclear.

Despite increased awareness, RLS remains underdiagnosed, especially in primary care settings [15]. Patients frequently present with sleep problems, mood changes, or non-specific sensory symptoms, and clinicians may not consistently apply the standard diagnostic criteria. Mild and moderate cases are often overlooked or misclassified, resulting in inadequate treatment. This has important consequences. Untreated RLS is associated with poor sleep, reduced daytime performance, lower work productivity, and a higher risk of depressive symptoms [16]. Associations with other conditions, such as cardiovascular disease, are less clear and require further study.

From a public health perspective, these findings highlight several priorities. Clinicians should actively consider RLS in high-risk groups, including older adults, women, and individuals with iron deficiency, chronic kidney disease, or pregnancy. Future studies should aim to improve diagnostic accuracy by combining clinical assessment with biological markers and long-term follow-up. In addition, RLS should be recognized as a neurological condition with a meaningful impact on health and daily functioning, rather than a minor or benign complaint.

A clearer understanding of RLS epidemiology will help reduce delays in diagnosis, improve patient care, and provide a more accurate estimate of its impact at the population level.

## 4. Clinical Presentation and Systemic Implications of RLS

**Figure 1:**
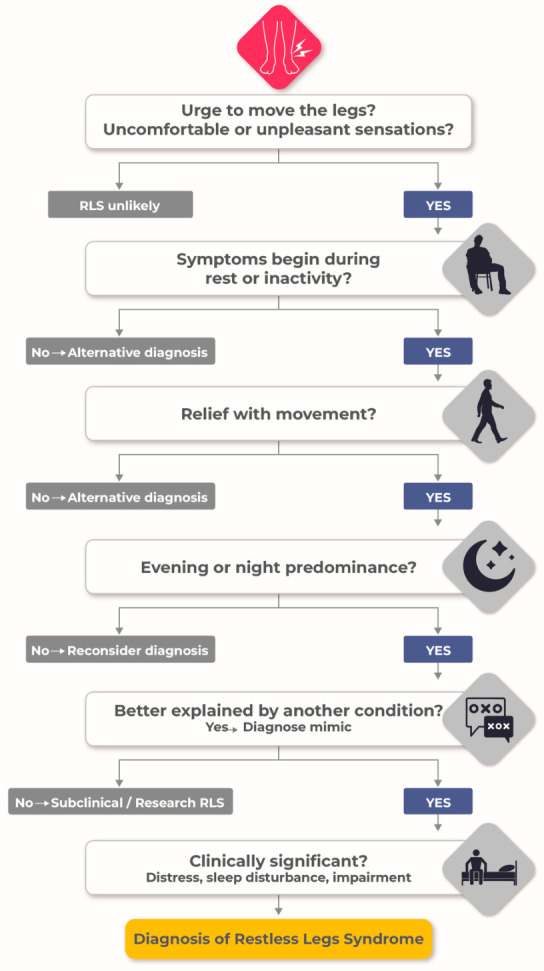
Diagnostic algorithm for RLS. The diagnostic framework for restless legs syndrome (RLS) follows a sequential, symptom-based pathway. Evaluation begins with the presence of an urge to move the legs, typically accompanied by uncomfortable or unpleasant sensations. Core diagnostic features include symptom onset or worsening during periods of rest or inactivity, partial or complete relief with movement, and a circadian pattern with predominance in the evening or night. The algorithm incorporates stepwise exclusion of alternative explanations, including leg cramps, positional discomfort, neuropathy, and other mimicking conditions, before confirming the diagnosis. Clinical significance, defined by distress, sleep disturbance, or functional impairment, is required for routine clinical diagnosis but may be omitted in selected research contexts when explicitly specified. The visual framework also reflects key modifiers that influence phenotypic expression, including extension of symptoms beyond the legs, variability in sensory perception, reduced responsiveness to movement in severe disease, and attenuation of circadian features in treatment-related augmentation. The coexistence of comorbid conditions, such as iron deficiency, chronic kidney disease, or pregnancy, does not preclude a diagnosis of RLS. Special considerations apply in pediatric populations, where symptom description may require age-appropriate interpretation.

RLS is defined clinically by a compelling urge to move the legs, often accompanied by unpleasant sensory phenomena, that emerges during rest, improves with movement, predominates in the evening or night, and cannot be explained by alternative conditions [17,18]. These criteria remain the foundation of diagnosis and underscore a critical principle: RLS is a clinical diagnosis grounded in circadian pattern, motor relief, and phenomenology rather than laboratory or electrophysiological findings (Figure 1).

### 4.1. Symptom Spectrum and Functional Burden

Although the legs are most commonly affected, symptoms may involve the thighs, feet, upper limbs, or, less frequently, the trunk. Presentation can be unilateral or bilateral. Severity ranges widely, from transient discomfort during prolonged immobility (e.g., long-haul travel) to near-continuous distress that renders stillness intolerable. In severe cases, relief with movement is incomplete, and the typical nocturnal predominance may blur as symptoms extend into daytime hours.

Sleep disruption is central to disease burden. Patients often seek care when symptoms occur at least twice weekly and cause moderate or greater distress [12,19]. In a survey of 551 individuals with RLS, 69% required at least 30 min to fall asleep, and 60% reported three or more nocturnal awakenings [19]. Objective polysomnographic data corroborate these findings. A 2022 meta-analysis of 31 case–control studies demonstrated that, compared with healthy controls, individuals with RLS had reduced total sleep time (358 vs. 386 min), lower sleep efficiency (75% vs. 84%), and increased wake time after sleep onset (81 vs. 54 min; all *p* < 0.001) [20].

Daytime sequelae are substantial. In a cohort of 416 patients, 32.2% reported excessive daytime sleepiness, 19.2% difficulty concentrating the following afternoon, and 40.1% interference with daily activities [12]. Nearly half (45.7%) identified sensory discomfort or pain as their most troubling symptom [12]. Sleep deprivation further exacerbates symptom intensity, creating a feed-forward cycle of hyperarousal and motor restlessness [21]. In a clinical context, these findings emphasize that RLS extends beyond a sensory–motor complaint and represents a disorder of sleep–wake regulation with measurable cognitive, behavioural, and functional consequences.

### 4.2. Neuropsychiatric Associations and Network Implications

RLS frequently coexists with mood and anxiety disorders. A 2024 systematic review and meta-analysis including 2039 individuals with RLS found a pooled prevalence of clinically significant depressive symptoms of 30.4% (95% CI 20.6–42.4%) [22]. Earlier observational data showed increased 12-month prevalence of panic disorder, generalized anxiety disorder, and major depression among patients with RLS compared with community controls (odds ratios ranging from 2.6 to 4.7) [23].

However, most available studies are cross-sectional, and causality cannot be inferred. The observed associations may reflect bidirectional interactions between sleep disruption, chronic sensory distress, and underlying neurobiological vulnerability. Neuroimaging findings suggest involvement of limbic–striatal circuits, supporting the hypothesis that affective and sensorimotor symptoms share partially overlapping network substrates, although these findings remain heterogeneous across studies.

### 4.3. Cardiovascular Associations: Signal, Confounding, and Uncertainty

Cardiovascular disease (CVD) represents another domain of interest. In a cross-sectional study of 3433 participants, CVD prevalence was 29.6% among individuals with RLS compared with 19.5% among those without RLS (adjusted odds ratio 2.07) [24]. Associations with hypertension are similarly dose-dependent. Among 65,544 women aged 41–58 years, hypertension prevalence increased from 21% in those without RLS to 33% in those reporting symptoms at least 15 times per month (adjusted odds ratio 1.41; *p* trend < 0.001) [25].

Mechanistic hypotheses include chronic sleep disruption, sympathetic activation linked to periodic limb movements of sleep (PLMS), and systemic inflammation [26,27]. However, these associations remain observational and may be confounded by factors such as comorbid conditions, medication exposure, and lifestyle variables. As such, a causal relationship between RLS and cardiovascular disease has not been definitively established. From a clinical perspective, these findings support consideration of cardiovascular risk assessment in patients with severe or chronic RLS, while acknowledging the current uncertainty regarding causality.

### 4.4. Periodic Limb Movements of Sleep: Motor Signature or Epiphenomenon?

PLMS, stereotyped, repetitive limb movements occurring at 15–30 s intervals during sleep, are detected by electromyography during polysomnography [28]. Approximately 30% of PLMS are associated with brief cortical arousals and are accompanied by transient increases in heart rate and blood pressure [29,30]. In patients with RLS who undergo polysomnography, PLMS are common and often regarded as the motor correlate of the disorder.

However, PLMS lack diagnostic specificity. They occur in congestive heart failure, multiple sclerosis, and in individuals receiving serotonergic reuptake inhibitors, and are frequently observed in healthy adults over 50 years of age [31,32]. In a community-based sample of 592 adults, 22.5% of those with frequent PLMS (>15/hour) reported RLS symptoms compared with 5.5% of those without frequent PLMS [33].

These findings suggest that PLMS may reflect a shared neurophysiological vulnerability, potentially related to altered excitability within motor and sleep-regulatory networks, rather than a disease-specific biomarker. Thus, while PLMS may provide supportive physiological information, they should not substitute for clinical criteria in diagnosis.

## 5. Assessment and Diagnosis: From Symptom Recognition to Mechanism-Informed Evaluation

RLS remains a clinical diagnosis based on established criteria, centred on four essential features: an urge to move the legs, worsening at rest, relief with movement, and circadian predominance in the evening or night. These criteria, as defined by international consensus groups, particularly the International Classification of Sleep Disorders, provide a robust, standardized, and clinically practical framework for diagnosis in routine care (Table 1). However, reliance on symptom-based criteria alone does not capture the disorder’s biological heterogeneity or distinguish between mechanistically distinct subtypes [34,35].

### 5.1. Clinical Evaluation and Differential Diagnosis

The diagnostic process begins with a detailed clinical history, including symptom timing, triggers, relieving factors, and functional impact. Particular attention should be given to the circadian pattern, as this remains a key discriminator from mimicking conditions (Table 2). Common differential diagnoses include peripheral neuropathy, nocturnal leg cramps, akathisia, positional discomfort, and anxiety-related restlessness. Distinguishing features include the characteristic circadian pattern and reproducible relief with movement, which are less prominent or absent in these conditions. Medication history is essential, as several agents, including antidepressants, antipsychotics, and antihistamines, may exacerbate or mimic RLS symptoms.

### 5.2. Laboratory Assessment and Iron Status

Assessment of iron status is a central component of evaluation. Current guidelines recommend measuring serum ferritin, transferrin saturation, and related indices to identify systemic iron deficiency [3,48]. However, a critical limitation is that peripheral iron measures do not reliably reflect central nervous system iron availability. As discussed in earlier sections, neuroimaging and CSF studies suggest that brain iron deficiency may occur despite normal serum ferritin levels. This creates a diagnostic–mechanistic gap: while central iron dysregulation is implicated in pathophysiology, clinically accessible biomarkers remain indirect and imperfect. Consequently, current thresholds (e.g., ferritin < 75–100 ng/mL) should be interpreted within a broader clinical context, rather than as absolute indicators of disease presence or absence [11,48].

### 5.3. Role of Polysomnography and Objective Testing

Polysomnography is not required for diagnosis but may be useful in selected cases, particularly when the diagnosis is uncertain or when coexisting sleep disorders are suspected [3]. Periodic limb movements of sleep (PLMS) are frequently observed in RLS but lack specificity and are insufficient for diagnosis [49]. Objective measures such as actigraphy and emerging digital phenotyping tools may provide complementary information on circadian patterns and symptom variability, although their role in routine practice remains to be defined.

### 5.4. Toward Mechanism-Informed Diagnostic Frameworks

The current diagnostic paradigm is predominantly phenomenological, which limits its ability to capture underlying biological diversity. Increasing evidence suggests that RLS may encompass multiple pathophysiological subtypes, including iron-driven, circadian-modulated, and hyperexcitability-dominant phenotypes [50]. A mechanism-informed approach would integrate clinical features with emerging biomarkers, including: (a) brain iron imaging (e.g., quantitative susceptibility mapping), (b) genetic risk profiling, (c) neurophysiological markers of excitability, and (d) digital assessment of circadian patterns. At present, however, these tools remain primarily research-based and are not standardized for clinical use.

### 5.5. Limitations and Future Directions

Several limitations constrain current diagnostic strategies. First, symptom-based criteria may lead to misclassification, particularly in mild or atypical cases. Second, available biomarkers lack sensitivity, specificity, and accessibility for routine implementation. Third, heterogeneity across patient populations complicates the identification of a single diagnostic threshold. Future diagnostic frameworks should aim to integrate multimodal data, clinical, biological, and digital, to enable stratified classification and personalized management. Importantly, any transition toward biomarker-informed diagnosis must be guided by rigorous validation, reproducibility, and clinical utility, rather than theoretical plausibility alone.

## 6. Risk Factors and Associated Conditions: Toward Mechanism-Informed Stratification

RLS frequently emerges in clinical contexts characterized by altered iron metabolism, physiological stress, or neurodegenerative vulnerability. Recognizing these associations is not merely epidemiological; it is central to a more biologically grounded diagnostic and therapeutic framework. However, many associations remain observational, and causal relationships should be interpreted with caution.

### 6.1. Iron Deficiency and Systemic Iron Dysregulation

Iron deficiency remains the most consistently replicated risk factor for RLS [51]. In a cross-sectional study of 251 individuals with iron-deficiency anemia presenting to a hematology practice, nearly one quarter (23.9%) reported RLS symptoms occurring at least twice weekly and associated with moderate-to-severe distress, as assessed by the 13-item Cambridge–Hopkins Restless Legs Syndrome Questionnaire [52]. These data underscore the bidirectional interface between hematology and neurology.

Importantly, iron-related vulnerability extends beyond overt anemia. More than 40% of patients with RLS who do not meet criteria for anemia nevertheless have iron indices within the therapeutic threshold for supplementation, defined as ferritin concentrations below 100 ng/mL, transferrin saturation below 20%, or both [11,48]. This observation reinforces a critical translational principle: peripheral iron measures do not reliably reflect central iron availability.

Neuroimaging studies using quantitative susceptibility mapping and related MRI approaches provide indirect evidence for a link between reduced brain iron and symptom severity [2,53], although variability across methodologies and populations should be acknowledged. In a clinical context, this creates a translational gap: although central iron deficiency is mechanistically implicated, treatment decisions still rely on peripheral biomarkers that may underestimate disease-relevant iron status.

### 6.2. Pregnancy: A Model of Reversible Vulnerability

Pregnancy represents a prototypical physiological stressor associated with transient RLS. A 2018 meta-analysis including more than 51,000 pregnant women reported a pooled RLS prevalence of 22% across gestation, increasing from 8% in the first trimester to 22% in the third trimester [54]. Approximately one in five affected women experience severe or very severe symptoms [54].

Notably, prevalence declines to approximately 4% postpartum, and roughly 75% of cases resolve within one month of delivery [52,55]. This temporal profile implicates dynamic changes in iron metabolism, hormonal fluctuations, particularly estrogen and progesterone, and dopaminergic modulation. Pregnancy-associated RLS provides a natural model of reversible vulnerability linked to systemic and central physiological shifts.

However, the relative contribution of iron deficiency versus hormonal and circadian factors remains incompletely resolved, and available data are largely observational. Nevertheless, it also signals long-term risk: women who experience RLS during pregnancy appear more likely to develop chronic RLS later in life. This trajectory underscores the need for early identification and follow-up rather than dismissal as a benign gestational phenomenon.

### 6.3. Opioid Withdrawal and Endogenous Opioid Deficiency

RLS prevalence may reach 50% during opioid withdrawal, including in the context of opioid use disorder or discontinuation after postsurgical analgesia [38,56]. This association aligns with emerging neurochemical evidence of reduced endogenous opioid activity in RLS and supports the hypothesis that dysregulated opioidergic signalling contributes to symptom generation [57]. However, distinguishing between causation and the unmasking of pre-existing vulnerability remains challenging, as withdrawal states may transiently exacerbate underlying network instability.

In a clinical context, patients presenting with new-onset RLS during opioid tapering require careful differentiation between transient withdrawal phenomena and persistent RLS. Conversely, in refractory RLS, the efficacy of low-dose opioids may reflect restoration of deficient neuromodulatory tone rather than nonspecific sedation. These observations support a broader, network-based neurochemical model.

### 6.4. Adrenergic Modulation and Hyperexcitability in RLS

Adrenergic mechanisms may represent an additional layer of neuromodulatory dysregulation in RLS. The locus coeruleus–noradrenergic system modulates arousal, nociceptive processing, and spinal cord excitability, and is closely integrated with dopaminergic and adenosinergic networks [58]. Experimental and clinical observations suggest that increased noradrenergic tone may contribute to sensory hyperexcitability and sleep fragmentation, both of which are central features of RLS. Pharmacological evidence provides indirect support for this hypothesis: agents that enhance noradrenergic signalling, such as certain antidepressants, have been associated with worsening of RLS symptoms [11], whereas α2-adrenergic modulation and related mechanisms targeted by α2δ ligands (e.g., gabapentinoids) may reduce central excitability and improve symptoms [11]. In addition, the frequent association between RLS and conditions characterized by heightened sympathetic activity supports a role for adrenergic dysregulation within a broader network model of neuromodulatory instability [59].

### 6.5. Chronic Kidney Disease and Peripheral Neuropathy

Among patients with end-stage kidney disease, pooled RLS prevalence is approximately 24% based on a 2024 meta-analysis encompassing over 12,000 individuals [36]. Mechanisms likely include chronic inflammation, iron dysregulation, and uraemia-related neurotoxicity. RLS in this population is associated with impaired sleep, reduced dialysis adherence, and diminished quality of life. Nevertheless, these associations may be influenced by multiple confounding factors, including comorbid conditions, medication exposure, and metabolic disturbances.

Peripheral neuropathy of diverse aetiologies, including diabetic and idiopathic forms, is also associated with increased RLS prevalence, estimated at 21.5% [39]. However, distinguishing neuropathic dysaesthesia from RLS remains challenging. The key clinical discriminator remains the circadian pattern of symptoms and relief with movement. Future work integrating quantitative sensory testing and neurophysiological biomarkers may improve diagnostic precision.

### 6.6. Neurological Comorbidities: Network Convergence

RLS is reported in approximately 27.5% of individuals with multiple sclerosis [40], 20% of those with Parkinson’s disease, particularly following initiation of dopaminergic therapy [42], and 16% of patients with obstructive sleep apnoea [47]. These associations highlight shared network vulnerabilities involving sensorimotor integration, sleep–wake regulation, and neuromodulatory balance. In Parkinson’s disease, distinguishing primary RLS from medication-related phenomena (e.g., augmentation or akathisia) is essential, as dopaminergic therapy can both treat and confound.

In multiple sclerosis, demyelinating lesions affecting spinal or subcortical pathways may contribute to symptom expression [60]. In obstructive sleep apnoea, sleep fragmentation and intermittent hypoxia may amplify central excitability. These comorbidities suggest that RLS is not an isolated entity but a manifestation of broader network instability under specific biological pressures. However, shared mechanisms remain incompletely defined, and heterogeneity across studies limits the ability to draw definitive conclusions. From a systems perspective, these conditions converge on common pathways involving iron handling, inflammation, circadian disruption, and excitatory–inhibitory imbalance, reinforcing the conceptualisation of RLS as a disorder of distributed neuromodulatory networks rather than a single-pathway disease.

## 7. Genetic Architecture and Developmental Vulnerability

Genetic predisposition contributes substantially to RLS risk. Approximately half of individuals with idiopathic RLS report a first-degree relative with the disorder [61], and twin studies estimate heritability as high as 70% [62], although SNP-based heritability derived from genome-wide association studies (GWAS) is more modest (~20%) [63]. A large meta-analysis encompassing 116,647 cases and over 1,546,466 controls identified 164 risk *loci* [63], underscoring the polygenic nature of the condition.

Among replicated loci, *MEIS1* remains the most robustly associated gene [64,65]. Beyond its established role in limb and nervous system development, new data suggest that *MEIS1* influences iron metabolism pathways and neuronal excitability, linking developmental programming with adult symptom expression [66]. Variants in BTBD9, implicated in iron homeostasis and dopaminergic regulation, further strengthen the biological connection between genetic susceptibility and iron-dependent neuronal function [41,67].

These genetic findings also support a network vulnerability model, in which early-life alterations in iron handling and synaptic regulation predispose distributed cortico–striatal circuits to later dysregulation under environmental or physiological stressors [63]. These discoveries move the field beyond a purely symptomatic definition of RLS. They suggest that early-life neurodevelopmental factors may prime cortico–striatal circuits for later vulnerability, particularly under conditions of fluctuating iron availability, hormonal change, or systemic inflammation.

## 8. Pathophysiology of RLS: From Iron Dysregulation to Network Dysfunction

RLS is best understood not as an isolated dopaminergic disorder, but as a systems-level condition in which iron biology, genetic susceptibility, and neuromodulatory imbalance converge within cortico–striatal–thalamic circuits. New evidence supports a shift from single-neurotransmitter models toward a network-based framework, in which multiple interacting systems contribute to symptom generation and clinical variability.

### 8.1. Brain Iron Dysregulation and Blood–Brain Barrier Function

Iron deficiency in the central nervous system is among the most reproducible biological findings in RLS. Notably, systemic iron indices are often within normal limits. Nevertheless, multiple modalities, including postmortem analyses [16], advanced MRI techniques sensitive to regional iron content [68], transcranial ultrasonography [69], and cerebrospinal fluid (CSF) studies [70], consistently demonstrate reduced iron concentrations in the substantia nigra and related networks.

Mechanistic work suggests that this discrepancy reflects impaired iron transport across the blood–brain barrier rather than peripheral deficiency alone. Altered expression of transferrin receptors, divalent metal transporter 1, and ferroportin in brain microvasculature and choroid plexus tissue supports a model of dysfunctional central iron handling [71].

Importantly, iron availability appears to modulate multiple neurotransmitter systems simultaneously [39]. Experimental and translational data suggest that reduced brain iron may impair adenosine A1 receptor expression and signalling, thereby disinhibiting glutamatergic transmission and altering dopaminergic tone [72]. This provides a mechanistic bridge between iron deficiency and network-level hyperexcitability, rather than a linear dopaminergic deficit model [73]. This observation has immediate clinical implications: serum ferritin is an imperfect surrogate for brain iron availability [11]. Consequently, treatment decisions based solely on peripheral indices may underestimate the extent of central deficiency. However, current clinical algorithms still rely on peripheral biomarkers due to the lack of validated central measures, reinforcing the rationale for intravenous iron therapy in selected patients, even in the absence of overt systemic iron depletion.

### 8.2. Dopaminergic Dynamics and Circadian Modulation

Neuroimaging studies using positron emission tomography and single-photon emission computed tomography have demonstrated increased presynaptic dopaminergic activity, particularly within the striatum [8,74]. Rather than indicating dopamine deficiency per se, these findings point to altered dopamine turnover or receptor sensitivity.

CSF analyses reveal enhanced diurnal variation in dopamine-related metabolites and cofactors in individuals with RLS, with greater evening–night fluctuations than in controls [75]. This circadian modulation may account for the hallmark temporal pattern of symptoms. Within a network framework, dopaminergic alterations are best interpreted as context-dependent adaptations to upstream modulatory changes, including iron availability and adenosinergic signalling, rather than as a primary isolated deficit [76].

It also provides a mechanistic explanation for the clinical phenomenon of augmentation during chronic dopaminergic therapy, a complication that reflects maladaptive plasticity rather than simple under-replacement [11,77]. Collectively, these findings caution against long-term dopaminergic management and support the increasing use of non-dopaminergic approaches as first-line therapy in moderate-to-severe disease.

### 8.3. Opioidergic and Glutamatergic Contributions

RLS pathophysiology extends beyond iron and dopamine. Evidence of reduced CSF β-endorphin concentrations in affected individuals, particularly those with painful symptoms, suggests endogenous opioid deficiency as a parallel mechanism [44,78]. This aligns with the clinical efficacy of low-dose opioids in refractory RLS and supports a broader neuromodulatory framework.

Alterations in glutamatergic signalling have also been reported, including increased excitatory drive in thalamocortical pathways [79]. Such findings are consistent with hyperarousal models and may explain the frequent coexistence of insomnia and periodic limb movements. A key integrative component of this network is the adenosinergic system. Reduced adenosine A1 receptor activity, potentially secondary to iron deficiency, has been proposed to disinhibit glutamatergic transmission and amplify cortical and subcortical excitability [11,77]. This mechanism provides a biologically plausible link between iron dysregulation, sleep disturbance, and motor restlessness and may represent a central node within the RLS network. Together, these interacting systems, iron metabolism, dopaminergic modulation, adenosinergic signalling, glutamatergic excitability, and opioidergic tone, support the conceptualization of RLS as a disorder of distributed neuromodulatory imbalance rather than a single-pathway disease.

## 9. Management of RLS: From Symptom Suppression to Mechanism-Based Care

Management of RLS has evolved substantially over the past decade. Nevertheless, practice often remains reactive, focused on short-term symptom relief rather than long-term disease modification. A modern therapeutic strategy should begin with correction of reversible contributors, prioritize iron repletion and non-dopaminergic agents, and anticipate the risks of augmentation and psychiatric sequelae. This shift reflects growing mechanistic insight and updated guideline recommendations [5,11].

### 9.1. Initial Management: Addressing Modifiable Drivers

The first therapeutic step is not pharmacological. Clinicians should identify and discontinue medications that exacerbate RLS, most notably centrally acting H1 antihistamines, serotonergic antidepressants, and dopamine antagonists [34,35]. Contributing medical conditions should be actively managed. RLS symptoms may resolve after kidney transplantation in patients previously treated with haemodialysis; in a small observational cohort, all 11 patients experienced symptom remission within 1–21 days of transplantation [6]. Treatment of obstructive sleep apnoea may also attenuate RLS severity [45].

Alcohol can worsen symptoms and should be limited or avoided [80]. Behavioural interventions, including lower-body resistance training, treadmill walking three times weekly, minimizing prolonged immobility, warm baths, and evening stretching, offer modest but clinically meaningful benefit in selected individuals [81,82]. These measures reinforce a broader principle: RLS is embedded within systemic physiology and sleep–wake regulation, not confined to isolated motor circuits.

### 9.2. Iron Supplementation: Foundational Therapy

Iron repletion is guideline-endorsed first-line treatment in patients with ferritin ≤ 100 ng/mL or transferrin saturation < 20% [83]. Some data suggest benefit at ferritin thresholds below 300 ng/mL in selected cases, reflecting the imperfect correlation between peripheral and central iron stores [84]. Oral ferrous sulfate (325–650 mg daily) is appropriate for patients with low ferritin (<75 ng/mL). However, absorption declines substantially at higher ferritin concentrations, and gastrointestinal adverse effects are common. Intravenous iron is recommended for individuals with ferritin levels of 75–100 ng/mL, poor oral tolerance, or an inadequate response [11]. Ferric carboxymaltose is the most extensively studied formulation. In two randomized controlled trials, 48% of participants treated with the intervention were rated as much or very much improved, compared with 18% receiving a placebo (*p* < 0.001) [11]. Symptomatic benefit may take 1–3 months to peak [85].

A comparative trial in iron-deficiency anemia with RLS reported similar 6-week efficacy between oral iron and intravenous ferumoxytol (75% vs. 68%; *p* = 0.55), though adverse events were more frequent with oral therapy (55% vs. 11%) [86]. To mitigate hepatic iron overload, intravenous iron should be avoided when ferritin exceeds 300 ng/mL or transferrin saturation exceeds 45%. The translational message is clear: iron therapy is not ancillary; it is disease-modifying in a biologically defined subgroup. Future directions include MRI-guided stratification and biomarkers of central iron handling.

### 9.3. Pharmacological Therapy: Individualized and Stratified

Treatment selection should be guided by symptom frequency, duration, and circadian timing. Intermittent symptoms may warrant as-needed therapy; persistent daily symptoms require scheduled evening dosing. Shared decision-making is essential (Table 3).

#### 9.3.1. Gabapentinoids (α2δ Ligands)

Gabapentin, pregabalin, and gabapentin enacarbil (the only FDA-approved agent in this class for RLS) are recommended as first-line daily therapy [5,11]. A 2025 meta-analysis reported that 73% of patients treated with gabapentin enacarbil were much or very much improved, compared with 39% with placebo (*n* = 1632; *p* < 0.001) [7,11]. Pregabalin demonstrated similar efficacy (69% vs. 43%; *n* = 493; *p* < 0.001).

Objective sleep parameters also improve. In a crossover trial, gabapentin increased total sleep time (6.0 vs. 5.5 h; *p* = 0.01) and sleep efficiency (84.7% vs. 74.9%; *p* < 0.001) [87], with parallel improvements in subjective sleep quality [88]. Gabapentinoids may simultaneously address comorbid insomnia, anxiety, and neuropathic pain. Adverse effects, such as somnolence, dizziness, gait instability, cognitive slowing, weight gain, and mood disturbance, limit tolerability in some patients [11]. Nevertheless, unlike dopamine agonists, gabapentinoids have not been associated with augmentation in long-term studies [1].

#### 9.3.2. Dopamine Agonists

Pramipexole, ropinirole, and rotigotine are FDA-approved and effective in the short term. Across multiple randomized trials, approximately 60–67% of treated patients achieved substantial improvement, compared with 43–56% of placebo-treated patients [11]. Dopamine agonists improve both subjective and polysomnographic sleep measures [89,90]. However, chronic daily use carries a significant risk of augmentation, iatrogenic worsening characterized by earlier symptom onset, increased severity, and spread to upper extremities [91]. Incidence is estimated at 7–10% annually [92]. Impulse control disorders occur in 10–20% of patients, particularly at higher doses [93].

Current guidelines, therefore, discourage routine long-term daily use of dopamine agonists as first-line therapy [11]. If used, doses should remain within conservative limits (pramipexole ≤ 0.5 mg; ropinirole ≤ 2.0 mg; rotigotine ≤ 3.0 mg daily). Intermittent low-dose levodopa or dopamine agonists may be appropriate for episodic symptoms (e.g., travel-related immobility). A network meta-analysis found comparable efficacy between gabapentinoids and dopaminergic agents [94], but the absence of augmentation risk supports preferential use of gabapentinoids as initial daily therapy.

#### 9.3.3. Low-Dose Opioids: Refractory and Augmented Disease

For medication-refractory RLS or established augmentation, low-dose opioids are guideline-supported [11]. In a 12-week randomized trial, prolonged-release oxycodone–naloxone led to substantial improvement in 67% of participants versus 35% with placebo (*p* < 0.001) [95]. Long-acting methadone (2.5–20 mg daily) or buprenorphine (0.5–6 mg daily), doses far below those used for chronic pain, are recommended for persistent symptoms exceeding 10 h per day [37,96]. Common adverse effects include constipation and sedation. Registry data suggest relatively stable dosing over five years, with minimal escalation in most patients [37]. Nonetheless, careful risk assessment is mandatory, and stigma surrounding opioid use must be addressed through transparent, evidence-based counselling.

### 9.4. Neuromodulation and Emerging Therapies

Neuromodulatory approaches are gaining traction as adjunctive strategies for treatment-refractory RLS, reflecting a broader shift toward circuit-level interventions. High-frequency peroneal nerve stimulation has demonstrated clinical benefit, with a randomized trial reporting that 45% of treated patients were rated as much or very much improved compared with 16% in the sham group (*p* < 0.001) [97]. In parallel, non-invasive brain stimulation techniques such as transcranial magnetic stimulation (TMS) are emerging as promising tools to modulate cortical excitability and sensorimotor integration. Evidence from controlled studies suggests that repetitive TMS targeting motor and prefrontal regions may reduce RLS symptom severity and improve sleep-related outcomes, likely by normalizing dysfunctional cortico-striatal and thalamocortical circuits [98]. Although still investigational, these approaches highlight the therapeutic potential of targeting distributed neural networks beyond traditional dopaminergic paradigms.

### 9.5. Management of Augmentation

Augmentation remains the most challenging therapeutic complication. Tapering dopamine agonists must proceed gradually (10–25% dose reduction every 2–4 weeks), often after introducing a gabapentinoid [5,96,99]. Abrupt reduction risks severe rebound symptoms and dopamine agonist withdrawal syndrome, characterized by anxiety, depression, and suicidality [100]. If gabapentinoids are ineffective or poorly tolerated, transition to low-dose long-acting opioids is often required. In a retrospective series of 63 patients with augmentation, 78% achieved substantial improvement, and 59% discontinued dopamine agonists within nine months [101]. Notably, there are no randomized trials directly comparing augmentation strategies, an important evidence gap.

## 10. Prognosis: Chronicity, Systemic Risk, and the Imperative for Long-Term Strategy

RLS is often responsive to therapy, but rarely trivial in its longitudinal trajectory. More than 60% of patients achieve at least a 50% reduction in symptom severity with gabapentinoids or dopaminergic agents [102]. Nevertheless, symptomatic improvement does not equate to disease resolution. Even with effective pharmacological treatment, many individuals continue to report residual restlessness, fragmented sleep, and coexisting mood and anxiety symptoms [37,103].

This partial response profile underscores a fundamental clinical reality: RLS is frequently chronic, fluctuating, and multisystem in its impact. Symptom severity varies over time and may worsen in response to depression, anxiety, immobility, pain, iron deficiency, or exposure to triggering medications. Age-related progression is common, particularly in individuals with severe baseline disease, although spontaneous remission can occur in milder cases [104].

### 10.1. Psychiatric Morbidity and Suicide Risk

Longitudinal data reveal important psychiatric implications. In a prospective cohort of 56,399 US women followed for six years, 3.6% of participants with RLS developed clinical depression compared with 2.2% of those without RLS (multivariate-adjusted relative risk 1.49, 95% CI 1.06–2.10) [101]. These findings align with contemporary meta-analyses demonstrating elevated depressive symptom burden in RLS populations [22].

More concerning is the association with self-harm. A US cohort study excluding individuals with prior suicide attempts, cardiovascular disease, or cancer reported incidence rates of suicide and self-harm of 0.35 per 1000 person-years among participants with RLS versus 0.11 among those without RLS [105]. After adjustment, including for depression, the hazard ratio was 2.66 (95% CI 1.70–4.15). Although observational, these data suggest that the neurobiological and sleep-related burden of RLS may extend beyond distress to measurable psychiatric risk. In a clinical context, routine screening for mood disorders and suicidal ideation is not ancillary but essential. Within the research field, the intersection between dopaminergic dysregulation, sleep fragmentation, and limbic circuitry warrants further mechanistic study.

### 10.2. Mortality and Cardiovascular Outcomes

Emerging evidence suggests that RLS may carry broader systemic implications. In a prospective cohort of 18,425 US men without diabetes, arthritis, or kidney failure at baseline, eight-year mortality was 24.8% among those with RLS compared with 14.6% among those without RLS (*p* < 0.001 after adjustment for age and comorbidities) [101]. Among patients with end-stage kidney disease, RLS has also been associated with reduced survival. In one study of 204 individuals receiving dialysis, one-year survival was 82% in those with RLS versus 91% in those without, and two-year survival was 62% versus 72%, respectively (*p* < 0.02 after adjustment) [106].

Although causality cannot be inferred, proposed mechanisms include chronic sleep disruption, autonomic activation associated with periodic limb movements, systemic inflammation, and shared cardiometabolic risk factors. These hypotheses are supported by accumulating evidence linking sleep disturbance to cardiovascular morbidity. Importantly, treatment may mitigate risk. A 2021 prospective cohort study with 3.4 years of follow-up, including 16,694 treated patients with RLS and 7505 untreated individuals, reported that pharmacological treatment (dopaminergics, gabapentinoids, or opioids) was associated with a 13% lower incidence of cardiovascular disease (95% CI 4–20%) after adjustment for demographic and clinical factors [107]. While observational, this finding suggests that effective symptom control, or the biological mechanisms targeted by treatment, may have systemic benefit.

## 11. A Network Model of Iron-Dependent Neuromodulatory Instability in RLS

RLS is unlikely to arise from dysfunction within a single transmitter pathway or from a purely descriptive clinical construct [11,16]. A more coherent account emerges from converging evidence across neuroimaging, genetics, pharmacology and clinical observation, which together point to instability across interacting neuromodulatory systems embedded within distributed cortico–striatal–thalamo–limbic networks [11,16].

Within this framework, brain iron is not a passive substrate but a central regulator of chemical reactivity and cellular stability [108]. Iron is tightly controlled across multiple biological scales [109]. At the vascular interface, transferrin-bound iron crosses the blood–brain barrier via receptor-mediated transport, after which it is distributed to neurons and glial cells [110]. Within cells, iron is buffered by ferritin to prevent uncontrolled redox activity, while a labile iron pool remains available for metabolic needs [111]. This balance is dynamic: too little bioavailable iron limits essential reactions, whereas poorly sequestered iron promotes oxidative stress.

At the chemical level, iron cycles between ferrous (Fe^2+^) and ferric (Fe^3+^) states, enabling electron transfer reactions that underpin oxidative phosphorylation, oxygen handling and enzymatic catalysis [112]. In mitochondria, iron–sulfur clusters and haem groups form the core of the electron transport chain, linking iron directly to cellular energy production [113]. Subtle disturbances in iron handling can therefore impair ATP generation, increase reactive oxygen species and alter neuronal resilience, particularly in metabolically demanding regions such as the basal ganglia [114].

Iron is also deeply embedded in neuromodulatory biology [115]. In dopaminergic neurons, it acts as an essential cofactor for tyrosine hydroxylase, thereby shaping dopamine synthesis at its rate-limiting step [116]. In parallel, iron influences dopamine storage and release by modulating vesicular function and synaptic metabolism [117]. Beyond dopamine, iron modulates adenosine receptor expression and signalling, constrains glutamatergic tone by supporting astrocytic metabolism and glutamate clearance, and may influence noradrenergic activity through its role in mitochondrial function and oxidative balance within locus coeruleus neurons [118].

Importantly, iron distribution in the brain is regionally specialized and developmentally regulated [119]. High concentrations are found in the substantia nigra, putamen and globus pallidus, regions central to motor and sensorimotor integration [120]. Oligodendrocytes, which require iron for myelin synthesis, represent a major cellular reservoir that links iron homeostasis to white matter integrity and conduction velocity [121]. Microglia also participate in iron handling, particularly during inflammation, when altered iron sequestration may further perturb local circuit function [122].

When iron availability is reduced or dysregulated, these tightly coordinated processes begin to lose coherence [123]. Enzymatic efficiency declines, mitochondrial function becomes less stable, and neurotransmitter systems drift out of their optimal operating range [124]. The consequence is not a uniform deficit but a state of chemical and physiological variability, in which neurons and circuits operate closer to the threshold of instability.

This shift has network-level consequences. Reduced iron-dependent control may lower inhibitory tone and amplify excitatory drive, altering the balance between cortical input, striatal processing and thalamic output [125]. Noradrenergic systems, particularly those linked to arousal, may further amplify this state [126]. Together, these changes favour a condition of heightened responsiveness and impaired damping, expressed clinically as sensory discomfort, motor restlessness and a strong circadian pattern of symptoms.

Such a model offers a parsimonious explanation for the clinical heterogeneity of RLS. The disorder spans a continuum from mild, intermittent symptoms to severe and treatment-resistant disease with marked sleep disruption and neuropsychiatric features [11]. A single-pathway explanation struggles to account for this range, whereas a network model allows variability to arise from differences in how and where instability develops within the system.

This perspective also exposes limitations in current diagnostic frameworks. Existing criteria rely on subjective symptom reports and remain largely disconnected from underlying biology [4]. Although clinically practical, this approach does not resolve mechanistic diversity, limits biological stratification and may contribute to variability in prevalence estimates [4]. Similarly, candidate biomarkers, including measures of brain iron and genetic risk, remain inconsistent across studies and are not yet standardized for routine use [127].

Therapeutic observations further support a network-based view. Dopaminergic treatments can provide short-term relief but often lead to augmentation, a paradoxical worsening that reflects maladaptive plasticity rather than correction of the underlying disturbance [11]. Other approaches, including α2δ ligands and iron supplementation, yield partial and variable benefit. These patterns suggest that current therapies act on downstream expressions of instability without restoring the chemical and circuit-level balance that sustains normal function [11].

From this standpoint, treatment resistance may reflect incomplete engagement of a distributed system rather than failure of a specific drug class [11]. Interventions that target a single pathway may transiently rebalance one node while leaving the broader network prone to drift.

**Figure 2:**
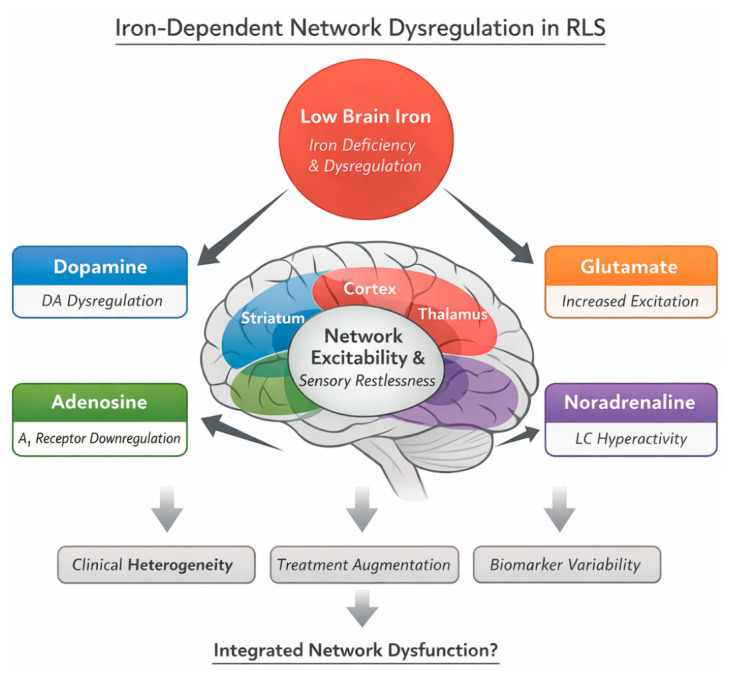
A hypothesis-driven network model of iron-dependent neuromodulatory instability in RLS. Here, brain iron is positioned as a central regulatory variable governing the stability of distributed neuromodulatory systems. This framework advances the hypothesis that even subtle reductions or dysregulation in iron availability, particularly within the tightly controlled Fe^2+^/Fe^3+^ redox couple, are sufficient to perturb core biochemical processes, including oxygen handling, mitochondrial respiration, and enzymatic catalysis. Iron acts as an essential cofactor for tyrosine hydroxylase in dopamine synthesis, participates in electron transfer within mitochondrial complexes, and contributes to redox buffering; thus, its imbalance may simultaneously impair neurotransmitter production and cellular energetics while promoting oxidative stress through aberrant redox cycling. Within this model, altered iron homeostasis destabilizes dopaminergic, adenosinergic, glutamatergic, and noradrenergic pathways, shifting cortico–striatal–thalamo–limbic networks toward increased excitability and reduced inhibitory tone. Dopaminergic dysfunction, adenosine A_1_ receptor downregulation, glutamatergic hyperactivity and locus coeruleus–mediated noradrenergic overactivity are therefore conceptualized not as isolated abnormalities, but as interdependent manifestations of a shared iron-sensitive regulatory axis. Their dynamic interactions across network nodes are proposed to shape sensory processing, motor output, and arousal regulation. This systems-level imbalance provides a mechanistic substrate for the core clinical features of restless legs syndrome, including sensory discomfort, motor restlessness, circadian symptom expression and sleep fragmentation. The model further suggests that clinical heterogeneity, biomarker variability and treatment-related augmentation arise from differential network susceptibility to iron-dependent perturbation, rather than from dysfunction within a single neurotransmitter pathway. Importantly, this framework is explicitly hypothesis-generating. The quantitative relationships linking iron chemistry and metabolism to neuromodulatory dynamics and large-scale network behaviour remain incompletely defined, and current evidence is heterogeneous. Future work integrating longitudinal imaging, molecular profiling and circuit-level interrogation will be required to determine whether iron-centred network instability can be reliably identified, biologically stratified and therapeutically targeted in restless legs syndrome.

This framework should be viewed as an integrative and testable hypothesis rather than a definitive account (Figure 2). Key elements, including the causal role of iron dysregulation, the direction of interactions among neuromodulatory systems and the relative contribution of each pathway, remain to be established. Nevertheless, the model generates clear predictions. Distinct biological subtypes of RLS should be identifiable through patterns of network organization across multimodal data. Treatment response should align more closely with underlying neuromodulatory profiles than with clinical presentation alone. Most importantly, strategies that restore stability at the network level rather than at the level of individual transmitters may achieve more durable control.

In this light, RLS can be viewed as a human model of how elemental chemistry shapes brain function across scales. A small shift in iron handling, in its transport, storage or redox state, may propagate from molecular reactions to cellular metabolism, from cellular metabolism to circuit dynamics, and from circuit dynamics to behaviour. Defining these links will require longitudinal and integrative approaches that combine neuroimaging, genetics, physiology, and real-world phenotyping, with sufficient resolution to capture iron biology as a dynamic, context-dependent process.

## 12. Discussion

RLS is at a conceptual crossroads. Historically framed as a dopamine-responsive sensorimotor disorder, it is increasingly recognized as a condition that cannot be fully explained by a single neurotransmitter deficit or by phenomenological clinical criteria alone. New evidence from neuroimaging, genetics, circadian biology, and pharmacology suggests that dopaminergic dysfunction occurs within a broader context of neuromodulatory imbalance embedded in distributed brain networks. However, this reconceptualization should be interpreted as an evolving framework rather than a definitive paradigm shift.

A central feature of the current evidence base is its marked heterogeneity. Neuroimaging studies, including quantitative susceptibility mapping and functional MRI, have yielded variable and sometimes conflicting findings across cohorts, likely reflecting differences in acquisition protocols, analytical strategies, and population characteristics [8,11,128]. Similarly, the clinical phenotype of RLS spans a broad spectrum, from intermittent sensory symptoms to severe, treatment-refractory disease with substantial sleep disruption and neuropsychiatric comorbidity [3,16,21,85,95]. Emerging biomarkers, including brain iron imaging and genetic risk profiling, show promise but remain limited by inconsistent reproducibility and lack of standardization. Taken together, these observations indicate that RLS is unlikely to represent a unitary disorder and instead reflects underlying biological diversity that current frameworks do not capture.

Within this context, the proposed network model of iron-dependent neuromodulatory instability provides a conceptual framework for integrating these disparate findings (Figure 2). This model posits that altered brain iron availability functions as a central modulator of distributed neuromodulatory systems, including dopaminergic, adenosinergic, glutamatergic, and noradrenergic pathways. Rather than acting as an isolated deficit, iron dysregulation is proposed to influence excitatory–inhibitory balance, neurotransmitter dynamics, and temporal regulation across cortico–striatal–thalamo–limbic circuits. In this framework, RLS emerges as a disorder of network instability, in which subtle perturbations in neuromodulatory interactions lead to altered excitability, sensory amplification, and circadian symptom expression.

This perspective offers a potential explanation for several unresolved features of RLS. Clinical heterogeneity may reflect differential involvement of network nodes and modulatory systems across individuals. Variability in neuroimaging findings may arise from differences in network state rather than solely from structural abnormalities. Similarly, the limited reproducibility of candidate biomarkers may reflect the dynamic, context-dependent nature of network dysfunction rather than the absence of a biological signal.

A central tension persists between therapeutic efficacy and biological understanding. Dopamine agonists improve symptoms in the short term, yet neuroimaging data do not support a simple dopamine-deficiency model [11]. Instead, imaging studies suggest altered presynaptic dopamine dynamics and receptor regulation rather than depletion [129]. The phenomenon of augmentation, iatrogenic worsening with chronic dopaminergic therapy, further challenges the deficiency paradigm and implicates maladaptive plasticity within striatal and circadian systems [11]. Within a network framework, these findings are more consistent with dysregulated neuromodulatory balance than with a primary neurotransmitter deficit.

Importantly, the network model provides a mechanistic context for these therapeutic limitations. Current treatments are predominantly symptomatic and target individual neurotransmitter systems. From a network perspective, such approaches may modulate downstream manifestations of instability without restoring system-level equilibrium. Augmentation and treatment resistance may therefore reflect incomplete modulation of distributed circuits rather than failure of specific pharmacological targets. This interpretation does not diminish the clinical utility of existing therapies but highlights their limitations in addressing underlying disease mechanisms.

Iron dysregulation remains a central, though not yet definitive, axis within this model. Convergent evidence from postmortem studies, cerebrospinal fluid analyses, and advanced MRI techniques suggests reduced iron availability in key brain regions, including the substantia nigra, even in the presence of normal peripheral indices [130]. Iron influences multiple neurobiological processes, including dopamine synthesis, mitochondrial function, myelin integrity, and adenosinergic signalling. In particular, downregulation of adenosine A_1_ receptors may disinhibit glutamatergic transmission and contribute to network hyperexcitability. However, direct causal links between iron restoration and normalization of network function remain unproven, and peripheral biomarkers do not reliably reflect central iron status.

Genetic findings further support a model of distributed vulnerability. Genome-wide association studies have identified numerous risk loci, including *MEIS1* and BTBD9, implicating pathways related to iron homeostasis, neurodevelopment, and dopaminergic regulation [131,132]. These data suggest that RLS may arise from an interaction between developmental susceptibility and later-life perturbations, including iron deficiency, hormonal changes, and systemic stressors [133]. Nevertheless, translating genetic findings into clinically meaningful stratification remains limited, and genotype–phenotype relationships remain incompletely defined.

Circadian biology represents an additional, underdeveloped dimension. RLS is characterized by temporally gated symptoms, with worsening in the evening and night [134]. Fluctuations in dopaminergic metabolites and cofactors across the circadian cycle have been observed [135], but the integration of circadian dynamics with iron metabolism and network excitability remains poorly understood. Incorporating temporal biology into network models will be essential to capture the disorder’s pathophysiology fully.

Beyond dopamine and iron, additional neuromodulatory systems contribute to the emerging picture. In a preprint, increased glutamatergic tone within thalamocortical pathways has been associated with hyper-arousal and sensory amplification [136]. Reduced endogenous opioid activity may underlie the efficacy of low-dose opioids in refractory cases. Adrenergic pathways, through locus coeruleus projections, may influence arousal, sensory gating, and spinal excitability [137]. However, the hierarchy and directionality of these interactions remain unclear, and it is not yet established whether these changes represent primary drivers or compensatory responses.

Clinically, a persistent paradox remains: many patients experience symptomatic improvement with available therapies, yet continue to report impaired sleep, mood disturbance, and functional limitations [11]. Associations with depression, self-harm, and cardiovascular outcomes have been reported, but causality remains uncertain and may reflect shared vulnerabilities rather than direct disease effects [133]. These observations further support the view that RLS extends beyond a simple sensorimotor disorder and involves broader network dysfunction.

Methodological limitations continue to constrain progress. Clinical trials rely heavily on subjective rating scales, while objective biomarkers remain underdeveloped. Diverse populations are underrepresented in genetic studies, and pediatric data are limited. Mechanistic studies integrating imaging, molecular markers, and longitudinal outcomes are scarce. As a narrative review, the present synthesis is also subject to inherent limitations, including potential selection bias and the absence of a formal risk-of-bias assessment.

The proposed network model should therefore be regarded as an integrative and testable hypothesis (Figure 2). It generates several predictions: that biologically distinct subtypes of RLS exist; that treatment response varies according to underlying neuromodulatory profiles; and that interventions targeting network-level stability may yield more durable outcomes. Testing these predictions will require longitudinal, multimodal studies integrating neuroimaging, genetics, neurophysiology, and digital phenotyping.

A central implication is that the current gap between clinical diagnosis and therapeutic outcomes may reflect a mismatch between phenomenological classification and underlying network biology. Bridging this gap will be essential to move from descriptive to mechanistically informed neurology.

## 13. Conclusions

RLS is increasingly understood as a disorder of iron-dependent neuromodulatory instability within distributed cortico–striatal–thalamo–limbic networks, extending beyond traditional dopamine-centric frameworks. Converging evidence from neuroimaging, genetics, and clinical studies supports a shift toward a network-based conceptualisation, although this shift remains incomplete. The network model proposed in this review (Figure 2) integrates current evidence into a unified, yet provisional, framework. It conceptualizes RLS not as a disorder of isolated neurotransmitter dysfunction, but as a condition arising from dysregulated interactions among multiple neuromodulatory systems influenced by iron availability, developmental factors, and circadian dynamics. This model provides a potential explanation for clinical heterogeneity, variability in biomarker findings, and the limitations of current therapies. However, this framework should be interpreted as an evolving, integrative hypothesis rather than a definitive mechanistic explanation. Substantial heterogeneity across studies, together with incomplete validation of candidate biomarkers and limited mechanistic data, underscores the need for cautious interpretation. Key elements, including the causal role of brain iron dysregulation, the interaction among neuromodulatory systems, and the identification of biologically meaningful subtypes, require rigorous empirical testing.

Future progress will depend on the integration of multimodal evidence, including advanced neuroimaging, genetic profiling, circadian biology, and digital phenotyping, within longitudinal study designs. Such approaches are essential to determine whether network-level dysfunction can be reliably identified, stratified, and therapeutically targeted. In this context, RLS offers a tractable human model to investigate how subtle metabolic and neuromodulatory perturbations destabilize neural networks governing movement, sleep, and affect. Advancing from symptomatic management toward mechanism-based intervention will require not only conceptual refinement but also robust validation across diverse populations.

## Figures and Tables

**Table 1:** Diagnostic framework for restless legs syndrome: clinical criteria, modifiers, and mechanistic interpretation.

Domain	Element	Clinical Signal	Interpretation (Network Level)	Evidence
Core criteria	Urge to move the legs	Compelling need to move → often with abnormal sensations	Sensorimotor integration disturbance → cortico–striatal dysregulation	[1,2,3,36,37]
	Rest-induced worsening	Symptoms ↑ during inactivity → sitting or lying	State-dependent network instability → reduced inhibitory tone	[36,38]
	Relief with movement	Movement → partial or complete symptom relief	Transient normalization of network excitability	[36,38]
	Circadian pattern	Evening/night predominance → symptoms ↑ at night	Circadian neuromodulation → dopamine and iron fluctuation	[1,2,3,36,37]
Exclusion	Alternative causes	Symptoms not explained by neuropathy, cramps, arthritis, or akathisia	Avoids diagnostic misclassification → ensures specificity	[36,38]
Clinical significance	Functional impact	Sleep disruption → ↓ sleep efficiency, ↑ awakenings	Sleep–wake instability → network hyperexcitability	[39,40,41]
	Daytime consequences	Daytime sleepiness → cognitive impairment → reduced function	Propagation from nocturnal disruption to daytime dysfunction	[41,42]
Phenotypic modifiers	Distribution	Legs → arms → trunk (in severe cases)	Spread of network involvement → disease progression	[38,41]
	Sensory variability	Dysesthesia may be subtle or absent	Risk of under-recognition → variable sensory processing	[41]
	Severe disease	Movement relief ↓ or incomplete	Persistent hyperexcitability → advanced dysregulation	[42]
	Treatment-related changes	Circadian pattern ↓ or lost during augmentation	Dopaminergic maladaptive plasticity → network destabilization	[11]
Contextual factors	Iron status	Ferritin ↓ (<75–100 ng/mL) → transferrin saturation ↓	Central iron deficiency → altered dopamine and glutamate signalling	[1,8,12,43]
	Pregnancy	Symptoms ↑ across trimesters → resolve postpartum	Hormonal + iron fluctuations → reversible vulnerability	[13,14]
	Chronic kidney disease	Increased prevalence of symptoms	Systemic inflammation → iron dysregulation	[44]
	Medications	Antidepressants, antipsychotics → symptoms ↑	Neurotransmitter imbalance → exacerbation of network instability	[45]
Associated physiology	Periodic limb movements during sleep	Repetitive movements → sleep fragmentation → ↑ heart rate	Marker of motor network excitability → not disease-specific	[23,24,26]
Diagnostic tools	Clinical history	Circadian pattern + movement relief → key diagnostic anchors	Core diagnostic framework remains clinical	[36,37]
	Laboratory assessment	Serum ferritin and iron indices	Peripheral markers → indirect proxy of brain iron	[12,43]
	Polysomnography	Detects limb movements → evaluates sleep structure	Adjunctive tool → not required for diagnosis	[23,24,25,26,27,46]
Emerging biomarkers	Brain iron imaging	Reduced iron signal in basal ganglia → MRI-based measures	Supports the iron-dependent network model	[1,8]
	Genetic profile	Risk loci (e.g., *MEIS1*, BTBD9)	Developmental–metabolic susceptibility	[9,10]
	Circadian phenotyping	Temporal variation in symptoms	Biological stratification → future precision medicine	[1,2,3]

**Table 2:** Distinguishing restless legs syndrome from common mimics: a clinically oriented framework.

Diagnostic Dimension	Restless Legs Syndrome	Neurological Mimics (Peripheral Neuropathy, Akathisia)	Musculoskeletal Mimics (Cramps, Arthritides)	Contextual Mimics (Anxiety, Positional Discomfort)
Core symptom	Urge to move → dysesthesia	Neuropathic pain → burning/electric Motor restlessness (akathisia)	Painful contraction → cramps Joint pain → stiffness	Inner unease (anxiety) Local pressure (posture)
Temporal pattern	Evening/night ↑ (hallmark)	No consistent circadian pattern	Episodic or activity-related	Variable → situational
Trigger	Rest/inactivity → symptoms ↑	Persistent (neuropathy) Drug exposure (akathisia)	Sudden (cramps) Load/movement (arthritis)	Stress or posture
Relief with movement	Sustained relief → key discriminator	Minimal (neuropathy) Temporary (akathisia)	Relief after stretching (cramps) Limited (arthritis)	Immediate repositioning or nonspecific
Neurological exam	Normal	Sensory deficits → reflex changes (neuropathy) Motor restlessness (akathisia)	Normal or joint inflammation signs	Normal
Clinical course	Chronic → fluctuating → circadian	Chronic or drug-related	Episodic or chronic structural	Situational or persistent
Pathophysiology	Iron ↓ → dopamine modulation ↓ → glutamate ↑ → network hyperexcitability	Peripheral nerve damage Dopamine receptor blockade	Muscle hyperexcitability Structural joint disease	Limbic hyperarousal Mechanical compression
Key discriminator	Circadian pattern + relief with movement	Objective neurological deficit or medication link	Pain-dominant → structural or muscular origin	Context-dependent → immediate resolution
Evidence	[1,2,3,23,24,25,26,27,36,37,46]	[36,37]	[36,37]	[17,47]

**Table 3:** Evidence-based treatments for Restless Legs Syndrome in adults.

Treatment Class/Agent	Typical RLS Dose Range	CGI-I Responder Difference vs. Placebo, % (95% CI)	IRLS Mean Difference vs. Placebo (95% CI)	AASM Recommendation (2025)	Common or Clinically Important Adverse Effects	GRADE (Certainty/Recommendation)
Strongly or Conditionally Recommended						
Gabapentinoids (α2δ Ligands)						
Gabapentin	300–3600 mg/d	—	−8.40 (−12.00 to −4.80)	Strong for	Somnolence (10–25%), dizziness (15–19%), cognitive disturbance, mood changes, weight gain	Moderate/Strong
Gabapentin enacarbil	600–1200 mg/d	34 (24 to 45)	−4.93 (−6.85 to −3.02)	Strong for	Similar to gabapentin	High/Strong
Pregabalin	75–600 mg/d	26 (17 to 34)	−4.81 (−6.21 to −3.42)	Strong for	Somnolence, dizziness, weight gain	High/Strong
Iron Therapy						
IV ferric carboxymaltose	1000–1500 mg (1–2 doses)	30 (16 to 44)	−7.43 (−11.89 to −2.97)	Strong for	Headache (12%), nausea (5%), hypophosphatemia, and rare hypersensitivity	High/Strong
IV ferumoxytol	1020 mg (1–2 doses)	—	−7.90 (−11.74 to −4.06)	Conditional for	Infusion reactions (rare)	Moderate/Conditional
IV low–molecular-weight iron dextran	1000 mg (single dose)	—	—	Conditional for	Rare anaphylaxis risk	Low–Moderate/Conditional
Oral ferrous sulfate	325–650 mg daily or alternate days	—	−9.20 (−15.23 to −3.17)	Conditional for	Constipation (12%), nausea (11%), diarrhea (8%)	Moderate/Conditional
Opioids (Refractory RLS)						
Oxycodone (prolonged-release)	5–40 mg/d	32 (21 to 43)	−5.60 (−8.18 to −3.02)	Conditional for	Constipation (47%), sedation, pruritus	Moderate–High/Conditional
Methadone	2.5–20 mg/d	—	—	Conditional for	Sedation, constipation	Low–Moderate/Conditional
Buprenorphine	0.5–6 mg/d	—	—	Conditional for	Fatigue, sweating, pruritus	Low–Moderate/Conditional
Nonpharmacologic						
Peroneal nerve stimulation	Device-based	34 (21 to 46)	−3.40 (−5.02 to −1.78)	Conditional for	Local discomfort (28%), skin irritation (9%)	Moderate/Conditional
Pharmacologic (Routine Daily Use)						
Dopaminergic Agents						
Levodopa	100–200 mg/d	—	—	Conditional against	Augmentation (7–10% annually), impulse control disorders (10–20%), nausea, somnolence	High (efficacy)/Conditional against
Pramipexole	0.125–0.5 mg/d	23 (18 to 27)	−4.86 (−6.20 to −3.52)	Conditional against		High (efficacy)/Conditional against
Ropinirole	0.25–2 mg/d	18 (13 to 23)	−3.98 (−5.36 to −2.60)	Conditional against		High (efficacy)/Conditional against
Rotigotine (transdermal)	1–3 mg/d	11 (0 to 22)	−4.67 (−6.18 to −3.16)	Conditional against	Application site reactions (34%)	High (efficacy)/Conditional against

GRADE reflects certainty of evidence (High, Moderate, Low) and direction/strength of recommendation. Dopaminergic agents demonstrate high short-term efficacy but are downgraded due to an increased risk of augmentation, whereas gabapentinoids and intravenous iron offer the most favourable balance between efficacy and long-term safety.

## Data Availability

No new data were created or analyzed in this study. Data sharing does not apply to this article.

## Abbreviations

The following abbreviations are used in this manuscript:

AASM	American Academy of Sleep Medicine
A1R	Adenosine A1 receptor
α2δ ligands	Alpha-2-delta ligands (e.g., gabapentinoids)
BBB	Blood–brain barrier
BTBD9	BTB domain-containing protein 9
CKD	Chronic kidney disease
CSF	Cerebrospinal fluid
CVD	Cardiovascular disease
fMRI	Functional magnetic resonance imaging
GWAS	Genome-wide association studies
*MEIS1*	Meis homeobox 1
MRI	Magnetic resonance imaging
NCS	Nerve conduction studies
PLMS	Periodic limb movements of sleep
QSM	Quantitative susceptibility mapping
RLS	Restless legs syndrome
